# Technologies for Detecting Falsified and Substandard Drugs in Low and Middle-Income Countries

**DOI:** 10.1371/journal.pone.0090601

**Published:** 2014-03-26

**Authors:** Stephanie Kovacs, Stephen E. Hawes, Stephen N. Maley, Emily Mosites, Ling Wong, Andy Stergachis

**Affiliations:** 1 Department of Epidemiology, University of Washington, Seattle, Washington, United States of America; 2 The Bill and Melinda Gates Foundation, Seattle, Washington, United States of America; 3 Department of Global Health, University of Washington, Seattle, Washington, United States of America; 4 Global Medicines Program, University of Washington, Seattle, Washington, United States of America; Johns Hopkins Bloomberg School of Public Health, United States of America

## Abstract

Falsified and substandard drugs are a global health problem, particularly in low- and middle-income countries (LMIC) that have weak pharmacovigilance and drug regulatory systems. Poor quality medicines have important health consequences, including the potential for treatment failure, development of antimicrobial resistance, and serious adverse drug reactions, increasing healthcare costs and undermining the public's confidence in healthcare systems. This article presents a review of the methods employed for the analysis of pharmaceutical formulations. Technologies for detecting substandard and falsified drugs were identified primarily through literature reviews. Key-informant interviews with experts augmented our methods when warranted. In order to aid comparisons, technologies were assigned a suitability score for use in LMIC ranging from 0–8. Scores measured the need for electricity, need for sample preparation, need for reagents, portability, level of training required, and speed of analysis. Technologies with higher scores were deemed the most feasible in LMICs. We categorized technologies that cost $10,000 USD or less as low cost, $10,000–100,000 USD as medium cost and those greater than $100,000 USD as high cost technologies (all prices are 2013 USD). This search strategy yielded information on 42 unique technologies. Five technologies were deemed both low cost and had feasibility scores between 6–8, and an additional four technologies had medium cost and high feasibility. Twelve technologies were deemed portable and therefore could be used in the field. Many technologies can aid in the detection of substandard and falsified drugs that vary from the simplest of checklists for packaging to the most complex mass spectrometry analyses. Although there is no single technology that can serve all the requirements of detecting falsified and substandard drugs, there is an opportunity to bifurcate the technologies into specific niches to address specific sections within the workflow process of detecting products.

## Introduction

The health and economic consequences of falsified and substandard drugs are most severe in low- and middle-income countries (LMIC) with weak pharmacovigilance and drug regulatory systems [1]. A systematic review and surveys have identified widespread problems with poor quality antimicrobial drugs and other essential medicines in Southeast Asia and sub-Saharan Africa [2]–[6]. Poor quality medicines have important health consequences, including the potential for treatment failure, the development of antimicrobial resistance, and serious adverse drug reactions, including death, all of which may result in lost economic activity and increasing healthcare costs and may undermine efforts to improve healthcare [7].

A variety of technologies from analytical chemistry and other scientific fields have been used to detect falsified and substandard drugs. These technologies vary considerably in characteristics that impact their appropriateness for use in LMIC. For example, the range of technologies includes inexpensive field assays as well as sophisticated laboratory instruments and methods. Furthermore, detection technologies differ in the type of data - qualitative and quantitative – provided about a sample medicines. Qualitative tests demonstrate the presence or absence of the specific active pharmaceutical ingredient (API) while quantitative tests ensure that the necessary API is present in the correct dosage. Technologies also differ in the amount of training required for technicians to use them; some are portable and require little training while others require sophisticated laboratory equipment and a high level of technical expertise, making them more or less appropriate in LMIC.

The need for technologies to detect falsified and substandard drugs in LMIC is best illustrated by the global fight against malaria. Globally, 228 million doses of artemisinin-based combination therapy (ACT), the most common treatment for malaria, are consumed annually [8], but studies have shown that up to 1/3 of all ACT medicines in Asia and sub-Saharan Africa are falsified or substandard [5], [9], [10]. Making detection technologies more accessible in LMICs where there is a large problem of falsified and substandard drugs is essential. In attempting to better define the problem of poor quality medicines, the Institute of Medicine of the National Academies noted that making detection technology more accessible in LMICs has an important role in combating falsified and substandard drugs [7]. To address this growing problem, the United States Pharmacopeia Convention (USP) and the United States Agency for International Development (USAID) created the joint program Promoting Quality Medicines in Developing Countries (PQM) to train and deploy technologies for detecting falsified and substandard drugs in developing countries [11]. The aim of this article is to review technologies for detecting falsified and substandard drugs and to compare the suitability of these technologies for use in LMICs.

## Methods

Technologies for detecting substandard and falsified drugs were identified through online literature searches, non-peer reviewed technical reports and other online information, and expert interviews. We first conducted a systematic review of the literature to identify technologies using the PRIMSA guidelines [12]. Literature searches were conducted using PubMed, Web of Science, and Google Scholar. Search terms for each database included: “Technologies Detecting Counterfeit Drugs”, “Technologies Detecting Substandard Drugs”, “Mass Spectrometry Counterfeit Drugs”, “Colorimetry Counterfeit”, “Gas Chromatography Counterfeit”, “Liquid Chromatography Counterfeit”. We captured any technology described as being used for detecting counterfeit, falsified or substandard drugs, for determining pharmacokinetic parameters, or if the technology could plausibly be used in counterfeit drug detection according to expert opinion provided by manufacturers, inventors and study authors. No time bounds were placed on searches in order to maximize the number of technologies captured, but we limited our search to the English language publications. Because many details of the technologies including information about costs and training and laboratory needs were not always available in the published literature, we conducted expert interviews with pharmaceutical industry leaders. Through our expert interviews, additional technologies were identified. In order ensure that all technologies were represented, and we had complete information on each technology, we expanded our search to non-peer reviewed technical reports and other online information.

We evaluated the suitability of each technology for use in low resource settings. This determination was based a priori on criteria including the need for laboratory supplies, the speed of analysis, the requirement of a power source, additional facility requirements, the levels of training required for operation, and cost. Technologies were grouped according to general purpose within a broader algorithm for detecting substandard and falsified products including visual inspection, chemical profiling and identification and quantification of active ingredients, and confirmation testing [13].

We compared the performance of technologies according to their published sensitivities and specificities for detecting substandard and falsified drugs. For some technologies, published sensitivities and specificities were unavailable, but the performance of the technology had been compared against another technology. Some technologies are still under development and it was not possible to define their performance. We classified technologies as having low, medium or high sensitivity and denoted technologies that were deemed gold standards where appropriate.

Each technology was assessed according to the need for sample preparation and requirements for additional laboratory supplies. Many technologies do not directly test solids such as tablets; instead, solid substances need to be dissolved into solution or heated to high temperatures and vaporized into a gas state, such as many mass spectrometry technologies. We also evaluated technologies for speed of analysis that we dichotomized as fast (less than 5 minutes a test) and slow. We categorized technologies according to whether they require an external electricity supply with consistent voltage, are battery-powered, or do not require electricity.

We categorized technologies as portable, requiring a basic laboratory or requiring a research laboratory. Facility requirements ranged from any basic laboratory bench to laboratories capable of safely storing flammable gases. In addition to facility requirements, we evaluated the level of skill required to operate the technology. Some technologies require a trained chemist to operate while others require only a basic understanding of chemistry.

In order to aid comparisons, technologies were assigned a “Suitability for use in LMIC” score ranging from 0–8. Technologies with higher suitability for use in LMIC scores were deemed the most feasible in LMIC contexts. Scores were given across each of the categories including 1 point for not requiring sample preparation, 1 point for not requiring laboratory supplies, 1 point for fast speed, 1 for not requiring electricity, 2 points for requiring minimal training and 1 point for requiring a laboratory technician, 2 points for being portable and 1 point for requiring a basic laboratory. We also evaluated the cost of the device as a one-time purchase, with the categories of low cost $10,000 or less, medium cost $10,000–100,000, and high cost $100,000 or greater (all prices are 2013 USD).

Another characteristic in which we compared technologies was their relative position in an independently developed standard workflow for detecting substandard and falsified drugs. The standard workflow was developed by the Counterfeit Drug Forensic Identification Network (CODFIN), a network of laboratories around the world that facilitates the testing of suspected substandard and falsified medicines [14]. The workflow starts with the inspection of packaging, followed by quantitative High Performance Liquid Chromatography (HPLC), Raman and Near-Infrared spectroscopy (NIR) and colorimetric tests for the correct API; dissolution testing is used to ensure the correct amount of the API is present. For drug samples that do not pass inspection using these tests, ambient mass spectrometry (MS) analysis is conducted to confirm the presence of a falsified drug. For drug samples that have been confirmed to be a falsified drug, isotope ratio MS, X-ray Diffraction (XRD), and nuclear magnetic resonance (NMR) are used to help identify the geographic source of production of the falsified medicines for forensic purposes [13].

## Results

Our search strategy identified 42 distinct technologies that are listed in Table 1 and 2 along with characteristics to facilitate comparisons. Thirty-six of these technologies are currently commercially available to detect falsified or counterfeit drugs, and six are either in development or could plausibly be redeployed for this purpose. Seventeen technologies were either portable or required only a basic laboratory making them ideal for use in the field. Ten technologies were identified through non-peer reviewed technical reports and other online information and five technologies were identified through key informant interviews. Figure S1 details the results of our search strategy.

**Table 1:** Comparison of technologies for detecting substandard or falsified drugs.

Technology	Purpose	Sample preparation needed	Performance	Laboratory supplies	Speed	Need electricity	Level of training Required	Facility Requirements	Device Price**	Suitability for use in LMIC score*
**Technologies for visual Inspection**										
**WHO Checklist** [15]	Examination of Packaging	No	Low	None	Fast	No	Pharmacist or healthcare provider	Portable	Low	6
**Nanotechnology with multidimensional atomic force microscopy** [16]	Examining of Packaging	Yes	High: watermarks are very difficult to counterfeit	Drug-specific label	Slow	Yes	Trained chemist	Basic Lab	Medium	1
**Technologies for detecting the presence of the correct API**										
**Colorimetry** [28]	Initial classification	Yes	High: specificity for all drugs: 0.94–1.00	Regents, UV light	Fast	No	Laboratory technician	Portable (with GPHF-MiniLab)	Low	4
**Refractometer** [27]	Dissolution	Yes	Moderate: Sensitivity ranged from 1–0.78 when combined with Colorimetry	Alcohol	Fast	No	Laboratory technician	Portable	Low	5
**Paper chromatography cards** [17]	Initial classification	Yes	High: Sensitivity ranged from 0.92–1 and specificity ranged from 0.88–1	Water	Fast	No	Laboratory technician	Portable	Low	6
**PharmaCheck** [7]	Chemical separation, quantification, identification	Yes	High: dissolution component detect within 3%	None	Fast	No	Laboratory technician	Portable	Low	6
**Capillary electrophoresis** [33]	Chemical separation, quantification, identification	Yes	Moderate: Quantification of API within 10% of true value	Reagents	Slow	Yes	Laboratory technician	Basic Laboratory	Medium	2
**Micellar Electrokinetic Capillary Chromatography (MECC)** [34]	Chemical separation, quantification, identification	No	Moderate: 98–102% recovery of analyte	Capillaries	Slow	Yes	Highly trained lab technician	Basic Laboratory	Low	3
**Gas chromatography- Flame Ionization Detector (FID)** [35]	Chemical separation, quantification, identification	No	High: GC-FID is considered to have lower analytical performance than GC-MS	GC columns	Slow	Yes	Highly trained lab technician	Research laboratory	Medium	2
**Headspace (sampler) gas chromatography**	Chemical separation, quantification, identification	No	High: Highly sensitive and specific	GC columns, headspace partition	Slow	Yes	Highly trained lab technician	Research Laboratory	Medium	2
**Anion-exchange chromatography** [36]	Chemical separation, quantification, identification	No	Unknown	-	Slow	Yes	Highly trained lab technician	Research Laboratory	Medium	2
**High performance liquid chromatography (HPLC)** [18]	Chemical separation, quantification, identification	Yes	Gold Standard	HPLC columns, pump, reagents, detector	Slow	Yes	Highly trained lab technician	Research Laboratory	Medium	1
**(Planar) thin layer chromatography (TLC) Speedy Apparatus** [20], [29]	Chemical separation, quantification, identification	Yes	Moderate: Can detect if tablet has below 85% or over 115% active ingredient	None	Fast	No (unless needed for UV lamp)	Minimal training	Portable	Low	5
**TLC -Fast Chemical Identification System (FCIS)** [21]	Chemical separation, quantification, identification	Yes	Unknown: Accuracy not assessed	Reagents for colorimetry, TLC set up	Fast	No	Minimal training	Portable	Unknown	5
**TLC-GPHF-MiniLab** [22]	Chemical separation, quantification, identification, Visual Inspection	Yes	Low: Only able to detect grossly substandard products (less than 80% of the active ingredient)	Reagents	Fast	Yes	Laboratory technician	Portable	Low	4
**Fourier Transform Infrared (FTIR) spectroscopy** [31]	Chemical Profiling	No	Moderate: only able to analyze the surface of the substance	No	Fast	NO	Laboratory technician	Portable	Medium	8
**Near infrared spectrometry** [22]	Chemical Profiling	No	High: Performs slightly better compared to Ramen	None	Fast	No	Laboratory technician	Portable	Medium	7
**Counterfeit Device #3** [19]	Chemical Profiling and Visual Inspection	No	Unknown	None	Fast	No	Minimal training	Portable	Low	8
**Raman spectrometry** [22]	Chemical Profiling	No	High: Less sensitive than Near Infrared MS	None	Fast	No	Laboratory technician	Portable	Medium	7
**Fluorescence spectroscopy** [37]	Chemical Profiling	Yes	High: Depends on the level of technological development	Solvents	Slow	Yes	Laboratory technician	N/A	Low	3
**NMR spectroscopy** [38]	Chemical Profiling	Yes	High: can be used to validate DESI and DART results	Solvents	Slow	Yes	Chemist	Research laboratory	Medium	0
**NQR spectroscopy** [24]	Chemical Profiling	No	Unknown	None	Slow	Yes	Laboratory technician	Portable	Unknown	4
**Powder X-ray diffraction** [39]	Chemical Profiling, Forensics	Yes	Unknown	None	Slow	Yes	Chemist	Research laboratory	High	1

* Suitability for use in LMIC scores were assigned as: 1 point for not requiring sample preparation, 1 point for not requiring laboratory supplies, 1 point for fast speed, 1 for not requiring electricity, 2 points for requiring minimal training and 1 point for requiring a laboratory technician, 2 points for being portable and 1 point for requiring a basic laboratory.

** Technologies that cost <$10,000 USD = low cost, $10,000–100,000 USD = medium cost and >$100,000 USD = high cost.

**Table 2:** Technologies for confirmation testing.

Technology	Purpose	Sample preparation needed	Performance	Laboratory supplies	Speed	Need electricity	Level of training Required	Facility Requirements	Device Price*	Suitability for use in LMIC score**
**Sample Preparation Techniques**										
**Liquid Chromatography**	Identification and quantification of APIs	Yes	Gold Standard	Solvents	Slow	Yes	Highly trained chemist	Research laboratory	High	0
**Gas Chromatography**	Identification and quantification of APIs	Yes	Gold Standard	Solvents	Slow	Yes	Chemist	Research Laboratory	High	0
**Plasma Pencil Atmospheric Mass Spectrometry (PPAMS)** [40]	Identification and quantification of APIs	Yes	Unknown	Solvents	Fast	Yes	Highly trained laboratory technician	Research Laboratory	Medium	4
**Flow Injection Gradient Ratio Standard Addition MS (FI-GRSA-MS)** [41]	Identification and quantification of APIs	Yes	Moderate: Validated against HPLC methods but not as sensitive	Solvents	Fast	Yes	Chemist	Research Laboratory	High	1
**Ionization Techniques**										
**Desorption Electrospray Ionization (DESI)** [42]	Identification and quantification of APIs	No	Moderate: Not as sensitive as other MS techniques	None	Fast	Yes	Highly trained laboratory technician	Research Laboratory	Medium	4
**Direct Analysis in Real Time (DART)** [43]	Identification and quantification of APIs	No	Moderate: Not as sensitive as other MS techniques	None	Fast	Yes	Highly trained chemist	Research Laboratory	Medium	3
**Atmospheric Pressure Solids Analysis Probe (ASAP)** [44]	Identification and quantification of APIs	No	Moderate: Comparable to DART and DESI	None	Fast	Yes	Chemist	Research Laboratory	Low	3
**Surface Acoustic Wave Nebulizer (SAWN)**	Identification and quantification of APIs	Yes	Moderate: More Sensitive than DART or DESI	Solvents	Fast	Yes	Chemist	Research Laboratory	High	1
**Direct Analysis in Real Time with SAWN**	Identification and quantification of APIs	Yes	Moderate: More sensitive than DART or DESI alone	Solvents	Fast	Yes	Chemist	Research Laboratory	High	1
**Matrix Assisted Laser Desorption Ionization (MALDI)**	Identification and quantification of APIs	Yes	Low: Not as sensitive as other MS techniques	Solvents	Slow	Yes	Chemist	Research Laboratory	High	0
**Inductively Coupled Plasma (ICP)** [29]	Identification and quantification of APIs	Yes	Moderate: More sensitive than DART and DESI	Solvents	Slow	Yes	Chemist	Research Laboratory	High	0
**Mass Detectors**										
**Drift tube ion mobility spectrometry (DTIMS)** [42]	Identification and quantification of APIs	Yes	Unknown compared to other MS devices	Unknown	Slow	Yes	Chemist	Research Laboratory	High	0
**Time of Flight (TOF)**	Identification and quantification of APIs	Yes	Moderate: Performs comparable to most other types of MS devices, but is not as sensitive as GC or LC MS	None	Slow	Yes	Chemist	Research Laboratory	High	1
**Quadrupole**	Identification and quantification of APIs	Yes	Moderate: Single quandrupole machines are not as sensitive as triple quads	Solvents	Slow	Yes	Chemist	Research Laboratory	High	0
**Triple Quadrupole**	Identification and quantification of APIs	Yes	High: More sensitive than single quads. Triple quads are considered one of the most specific types of MS devices.	Solvents	Slow	Yes	Chemist	Research Laboratory	High	0
**Ion Traps**	Identification and quantification of APIs	Yes	High: Very sensitive and very specific	Solvents	Slow	Yes	Chemist	Research Laboratory	High	0
**OrbiTrap**	Identification and quantification of APIs	Yes	Gold Standard: Very sensitive, used as a gold standard for MS devices	Solvents	Slow	Yes	Chemist	Research Laboratory	High	0
**Portable GC/MS** ∧	Identification and quantification of APIs	No, not if combined with headspace GC	High: Performs as well as laboratory based GC/MS systems	Headspace device	Slow	No	Highly trained laboratory technician	Portable	High	4
**Technologies for Forensic Testing**										
**Isotope Ratio Mass Spectrometry** [45]	Linking counterfeit samples	Yes	High: Very specific, used in forensic applications	Solvents	Slow	Yes	Highly trained chemist	Research laboratory	High	0

*Technologies that cost <$10,000 USD = low cost, $10,000–100,000 USD = medium cost and >$100,000 USD = high cost.

** Suitability for use in LMIC scores were assigned as: 1 point for not requiring sample preparation, 1 point for not requiring laboratory supplies, 1 point for fast speed, 1 for not requiring electricity, 2 points for requiring minimal training and 1 point for requiring a laboratory technician, 2 points for being portable and 1 point for requiring a basic laboratory.

∧ GC-MS represents the combination of a sample preparation technology, ionization technology and mass detector which is portable. This is the only mass detector combination that is portable.

### Technologies for visual inspection

Visual inspection of packaging is the first step in detecting a substandard or falsified drug in the CODFIN workflow [13]. We identified two technologies that aid in visual detection: the WHO Checklist and nanotechnology with multidimensional atomic force microscopy, which are as far apart as is possible in price and suitability for use in LMIC. The WHO checklist is a free, accessible checklist that aids in the visual detection of falsified and substandard drugs and can be easily used by healthcare workers, pharmacy and laboratory technicians with minimal training [15]. In contrast, an atomic force microscopy instrument costs approximately $100,000, and requires a climate-controlled facility and a highly trained chemist to operate. Atomic force microscopy is used to detect counterfeits by reading molecular watermarks imprinted during authentic production of medications [16]. Two additional technologies, the GPHF-MiniLab and the Counterfeit Detection Device #3 (CD3), have the capability to perform visual inspections of labels and packaging in addition to their primary function of detecting the correct active pharmaceutical ingredient (API). Although visual inspection is an important first step, many counterfeiters and producers of falsified pharmaceuticals use techniques that can evade sophisticated visual tests [7].

### Technologies for detecting the presence of the correct API

Identifying the presence of the correct API is the next step in the CODFIN workflow. Technologies that can be used to detect the correct API range in price from less than $1 for a single paper chromatography test to $50,000 USD for a Raman or NIR portable device, and range in feasibility from being able to detect the correct API in the field within seconds to technologies that require research laboratory space, highly trained staff and take up to 20 minutes to run a sample [17], [18]. We compare technologies that can be used to detect the correct API in Table 3.

**Table 3:** Comparison of technologies for detecting the presence of the correct API.

Suitability for use in LMIC*	Low Cost**	Medium Cost	High Cost
High	CD3, Paper Chromatography, TLC Speedy Apparatus, TLC-FCIS PharmaCheck	NIR Spectroscopy, Raman Spectroscopy, FTIR Spectroscopy	
Medium	MECC, TLC-GPHF-MiniLab, Colorimetry, Refractrometry	Capillary electrophoresis	
Low		Nanotechnology, Gas Chromatography, Flame Ionization Detector (FID), Anion-exchange chromatography, HPLC, NMR Spectroscopy	Powder X-Ray Diffraction

* Suitability for use in LMIC scores were assigned as: 1 point for not requiring sample preparation, 1 point for not requiring laboratory supplies, 1 point for fast speed, 1 for not requiring electricity, 2 points for requiring minimal training and 1 point for requiring a laboratory technician, 2 points for being portable and 1 point for requiring a basic laboratory. High scores = 6–8, medium scores = 3–5, low scores = 0–2.

** Technologies that cost <$10,000 USD = low cost, $10,000–100,000 USD = medium cost and >$100,000 USD = high cost.

Counterfeit Device #3 (CD3), Micellar Electrokinetic Capillary Chromatography (MECC), Thin Layer Chromatography (TLC)-Fast Chemical Identification System (FCIS), Near infrared (NIR), Fourier transform infrared (FTIR), and High performance liquid chromatography (HPLC).

There is a divide in technologies for detecting the presence of the correct API between those that require a laboratory and those that can be taken into the field. Portable technologies include Raman and near infrared (NIR) light spectroscopy, the Counterfeit Detection Device #3 developed by the FDA, Nuclear Quadrapole Resonance (NQR) spectroscopy, and thin layer chromatography techniques such as Fast Chemical Identification System (FCIS), the GPHF-MiniLab and Speedy Apparatus, as well as paper chromatography test cards. In addition, mobile labs have been used by the Chinese government to detect counterfeit drugs in the field which place the GPHF-MiniLab in a van enabling easier transportation [7]. Portable technologies are particularly beneficial to combatting substandard and falsified products because they allow for testing to occur at ports, pharmacies, or other gateways to the market.

### Portable technologies for detecting the correct API that require no sample preparation

Low cost technologies that are most suitable for use in LMIC include: Counterfeit Detection Device #3 (CD3), paper chromatography test cards, TLC Speedy Apparatus, TLC-Fast Chemical Identification System, and PharmaCheck device [7], [17], [19]–[21]. Among these technologies, the CD3 stands out for its reported ease of use [19]. With an anticipated unit cost less than Raman and NIR portable devices and no need for additional supplies, the CD3 will likely be inexpensive and requires no sample preparation. The CD3 is portable, battery powered, and reportedly does not require extensive training to operate [19]. However, the sensitivity and specificity of the CD3 have not been reported. Other portable devices that do not require sample preparation include Raman, NIR, and FTIR spectroscopy, and are all options for detecting the correct API that are suitable for use in LMIC. These technologies are portable, testing is relatively fast, and they require no extra supplies. Raman, NIR and FTIR can be used in the field by a technician with minimal training. These devices cost approximately $50,000 [22]. The limitation of CD3, X-ray Diffraction, Raman, and NIR portable devices is that they depend on the use of reference libraries of pharmaceuticals to identify falsified and substandard products. These libraries must be routinely updated when new generics or new compounds come to market, which may limit their feasibility. X-ray diffraction is currently only used in the laboratory settings, but has been adapted to field use for mining operations and is in development for use for field detection of falsified medicine [23]. Lastly, a portable NQR device that requires no sample preparation is under development at the Kings College London [24]. As for the performance of the above mentioned devices, Raman and NIR have been shown to have high performance with NIR performing slightly better than Raman [22]. NIR can distinguish differences as small as 2.5% of the expected API content [25]. The United States Food and Drug administration requires that the API be within 5% (95–105%) of the advertised dosage [26].

### Portable technologies for detecting the correct API that require sample preparation

Portable technologies for detecting the correct API that require sample preparation include the GPHF-MiniLab, Speedy TLC and Fast Chemical Identification System (FCIS), PharmaCheck, refractometers and paper chromatography test cards. These technologies range in price from $1 per test for paper test cards to $3780 per unit for the GPHF-MiniLab, which can perform both TLC and disintegration tests [27], [28]. Because these technologies require sample preparation, they also consume reagents to prepare samples and importantly, the sample is destroyed through testing. Performance of the technologies is dependent on the training of the technician as well as the technology. Thin layer chromatography (TLC) is able to determine whether a tablet is within 85%–115% of the expect amount of the API [20], [29]. GPHF-MiniLab has been the preferred technology for detecting substandard and falsified drugs in LMIC. It is produced by the Global Health Pharma Fund (GHPF) to be an easy to use and inexpensive set of test kits for testing drugs in the field [30]. An assessment of the GPHF-MiniLab found that it could only identify grossly substandard products, i.e. those that were <80% of the expected API [22]. While these technologies are less expensive than Raman and NIR, their sensitivities and specificities are also lower.

Newer technologies such as paper chromatography test cards and PharmaCheck may fill gaps left by technologies such as GPHF-MiniLab and handheld devices that are generally too expensive or difficult to use at points of care. Paper chromatography test cards developed by a team at the University of Notre Dame use chromatography-based methods to quickly determine whether the correct API is present and whether inappropriate fillers such as chalk, talc, or starch are present for commonly used antibiotics and tuberculosis medications. Because the tests are designed to be inexpensive (less than $1 per test), they could be used at hospitals, pharmacies and drug shops to test products before dispensing them to patients [17]. PharmaCheck is a device under development by a team at Boston University and uses fluorescent dissolution to perform quantitative tests on antibiotic and anti-malarial drugs. The anticipated price, as well as the sensitivity and specificity are not yet available, but PharmaCheck is designed to be inexpensive and easy to use in the field [7].

### Technologies for detecting the correct API that require a laboratory

Technologies for detecting the correct API that require a laboratory setting include: capillary electrophoresis, micellar electrokinetic capillary chromatography (MECC), gas chromatography, anion exchange chromatography, high performance liquid chromatography (HPLC), Fluorescence spectroscopy, nuclear magnetic resonance (NMR) and powder X-ray diffraction. Of these laboratory-based technologies, HPLC represents the gold standard for chemical separation, quantification and identification [31]. HPLC requires sample preparation and consumes reagents and electricity, and requires a highly trained technician to operate. In addition, an HPLC instrument costs approximately $50,000. Within the CODFIN workflow, HPLC is the first test conducted on samples when they arrive at the laboratory [13].

According to the CODFIN workflow, any sample indicated as being of poor quality requires further identification and quantification of the API to confirm the presence of a substandard or falsified drug [13]. Mass spectrometry (MS) techniques can be used for confirmatory testing of any product deemed suspect through examination of packaging, spectroscopy, and chromatography techniques. MS is used for the identification and quantification of APIs, expedients, and adulterants. Mass spectrometers are expensive devices requiring trained personnel and well-equipped laboratories. Mass spectrometers are composed of three parts: an ion source, mass detector, and analyzer. In addition, mass spectrometry requires some form of sample preparation. Techniques such as gas chromatography and liquid chromatography can be used as forms of sample preparation for MS analyses. Technological advances for detecting substandard and falsified drugs have been focused primarily on improving the ion source. For example Direct Analysis in Real Time (DART) allows for the analysis of solid substances without sample preparation. Different types of sample preparations and ion sources can be matched with different types of mass detectors to create new MS devices. For example, DART ion source can be paired with Time of Flight (TOF) mass detector or with a quadrupole detector.

### Technologies for confirmatory testing

Table 2 compares technologies for confirmation testing. All of these technologies can be used in MS analyses. Currently, there are no portable, cheap devices for confirmation testing. Devices such as Direct Analysis in Real Time (DART) and Desorption Electronspray Ionization (DESI) are two ion sources that can be added to most MS devices to allow for direct analysis of solid samples requiring no solvents or sample preparation. Both devices cost approximately $50,000 USD and can be combined with Time of Flight or quadrupole MS devices [32]. DESI devices are able to provide information about the spatial distribution of components in the sample [31]. Both of these devices play a critical role in the CODFIN workflow, confirming the presence of a falsified API first detected by Raman, NIR or colorimetric testing [13].

The gold standard and most sensitive MS devices are gas chromatography (GC) MS and HPLC-MS devices. These instruments typically cost $350,000–375,000 USD. They require sample preparation and solvents, and must be housed in a laboratory that can handle flammable gasses. GC-MS devices require highly trained chemists to operate. A portable version of the GC-MS device has been developed, but has yet to be used for detection of substandard and falsified drugs. A currently available device that can be used in the field costs $175,000 USD, but requires an electrical supply and is only used for environmental sampling. These highly sensitive and expensive devices are suitable for use at a national reference laboratory for confirmation testing of falsified and substandard drugs identified in the field.

### Technologies for forensic testing

The final step in the CODFIN workflow is forensic analysis of confirmed substandard or falsified drugs to determine the origin of manufacturing [13]. Isotope ratio mass spectrometry (IRMS) is able to provide linkages between substandard and falsified samples and source materials [32]. IRMS can be completed using any high resolution mass detectors, but time of flight and OrbiTrap are the most commonly used. In addition, XRD can be used to detect minerals such as talc and calcite that are often used by counterfeiters as binding agents for pills and tablets and provide clues as to where the falsified medicine was manufactured [13]. Forensic analyses most likely will only take place in reference laboratories as part of criminal investigations. These technologies are costly and required highly trained personnel. They form the critical last step in investigating substandard and falsified medicines and can lead to the identification and halting of criminal practices.

## Discussion

We identified 42 technologies that can aid in the detection of substandard and falsified drugs. These technologies range from simple of checklists for evaluating packaging to complex analytical chemistry for fingerprinting the source of a falsified drug. Given the extensive list of options, matching the best technology for each position in the workflow for detecting falsified and substandard drugs requires a comparison of the performance and requirements of each technology. The use of the technologies in LMIC adds additional considerations, such as low cost, portability, and no requirement of sample preparation. In this review, we have provided a broad overview of the technologies used to detect counterfeit and substandard drugs, and to highlight those technologies most suitable for use in LMIC.

The most important considerations in choosing the appropriate technology for detecting counterfeit and substandard drugs are the testing site and the purpose for testing. Factors related to the testing site include whether there is a consistent electrical supply, what level of training the staff have, and if the purpose of the testing is either screening or confirmation. For regulatory authorities, importers of drugs, and others involved in pharmaceutical supply chains that wish to ensure that large batches of medicine are genuine, devices such as the CD3, Ramen and NIR are appropriate. These technologies do not destroy the product and can be used quickly by customs officers at ports. Government agencies and donor organizations may be better financed to afford the price of Ramen and NIR technologies, and the advent of the CD3 device should make these technologies more affordable for LMIC. In addition, these devices will ultimately have a lower cost per test as they require no reagents or laboratory facilities, and they can test hundreds of drugs within a day. For healthcare workers and others working to ensure that the drugs given to a patient are genuine, technologies for visual inspection approaches such as the WHO check list are a useful first step, but is not sufficient to identify falsified and substandard products. Additional technologies such as paper chromatography, PharmaCheck, and GPHF-MiniLab may be required. These technologies allow clinicians to distinguish between drug resistance and treatment failure due to the organism or treatment failure due to a falsified or substandard drug. In these settings, cheap, highly feasible tests that require little training and supplies are essential.

Highly sensitive technologies such as DART or DESI, HPLC-MS and GC-MS are best suited for national reference laboratories for confirmation testing of field-identified falsified and substandard drugs. Given the high costs of these devices, and their facility and technical requirements for operation, very few laboratories in LMIC will be equipped for their use. Furthermore, the reagents and gasses needed for these devices can be difficult to procure. Nonetheless with national governments and donor organizations spending money on antibiotics, anti-malarials, and other drugs, ensuring the safety and efficacy of these drugs is critical. For example, globally $1.6 billion was spent on malaria control in 2010 including 228 million doses of ACT [8]. National reference laboratories are also the best for forensic analyses by isotope ratio MS and other technologies to identify the geographic source of production of falsified and substandard products.

Although this paper presents a systematic review of technologies for the detection of fake and substandard drugs, it has several limitations. First, information about the performance of technologies under development was not always available, and therefore this paper presents results from non-peer reviewed technical reports, other online information, and key informant interviews making it difficult to reproduce our results. In addition, there is no gold standard against which to compare all other technologies for detecting substandard and falsified drugs. The development of new technologies for detection of falsified and substandard drugs is a fast moving field, and new technologies may not have been included in our review. Despite these limitations, this paper serves as a framework for evaluating technologies and their suitability for use in LMIC.

The illegal enterprise of making falsified and substandard drugs is considered to be large [7]. The need for technologies to detect these drugs and save lives is paramount. Technologies under development such as the CD3 device, PharmaCheck, paper test cards and portable NQR devices offer the prospect of bringing less expensive and more sensitive technologies to the field where the need is greatest. Key in the fight against falsified and substandard drugs will be technologies that can easily and cheaply distinguish a falsified drug from a real drug regardless of the sophistication of the counterfeiter's methods and to ensure that drugs have not degraded through poor storage and handling, or have a lower than labeled dose of the API. Technologies alone will not solve the problem. Well-trained people to use these technologies, legal frameworks that remove the incentives for producing and distributing the drugs, and thoughtful well-designed screening systems will be needed to detect substandard and falsified drugs and ultimately save lives.

## Supporting Information

Figure S1 **Results from Sysematic Search.**
(TIFF)

Checklist S1 **PRISMA Checklist.**
(DOCX)
