# Supplementary figures and images for: Technologies for Detecting Falsified and Substandard Drugs in Low and Middle-Income Countries
Source: PLoS One. 2014 Mar 26;9(3):e90601. doi: 10.1371/journal.pone.0090601 (PMC3966738; doi:10.1371/journal.pone.0090601)

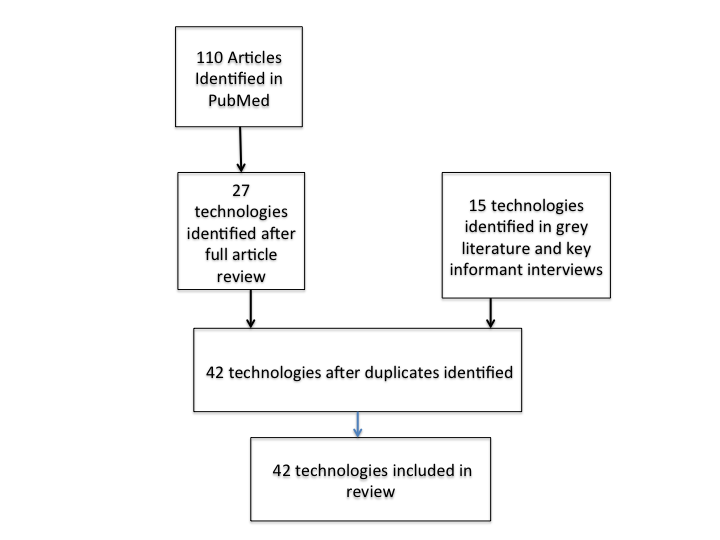

Supplement: Figure S1 — Results from Sysematic Search. (TIFF) [file pone.0090601.s001.tif]
